# Association of glycemic variability and time in range with lipid profile in type 1 diabetes

**DOI:** 10.1007/s12020-023-03464-x

**Published:** 2023-12-05

**Authors:** Mariana Salsa-Castelo, Celestino Neves, João Sérgio Neves, Davide Carvalho

**Affiliations:** 1 Faculty of Medicine, University of Porto, Porto, Portugal; 2 Department of Endocrinology, Diabetes and Metabolism, Centro Hospitalar Universitário de São João, Porto, Portugal; 3 Departament of Surgery and Physiology, Cardiovascular Investigation Unit, Faculty of Medicine, University of Porto, Porto, Portugal; 4 Instituto de Investigação e Inovação da Saúde, University of Porto, Porto, Portugal

**Keywords:** Type 1 Diabetes, Dyslipidemia, Continuous glucose monitoring, Time in Range, Coefficient of Variability

## Abstract

**Purpose:**

Hyperglycemia is associated with abnormalities of lipoproteins. The aim of this study was to analyze, in patients with Type 1 Diabetes (T1D), the association of glycemic control with lipid profile, focusing on glycemic variability and time in range obtained from Continuous Glucose Monitoring (CGM).

**Methods:**

We performed a retrospective cohort in patients with T1D. We analyzed clinical parameters, HbA1c, CGM and lipid profile in two moments 6 to 18 months apart. We evaluated the association of HbA1c and CGM metrics with lipid profile in cross-sectional (*n* = 242) and longitudinal (*n* = 90) analyses.

**Results:**

The mean age of the study population was 36.6 ± 12.6 years, 51.7% were male, and the mean diabetes duration was 16.8 ± 10.3 years. In the cross-sectional analysis, higher HbA1c, higher glucose management indicator (GMI), higher time above range and lower time in range were associated with higher triglyceride levels. In the longitudinal analysis, an increase in time below range was associated with a decrease of HDL cholesterol. In both analyses, an increase in the coefficient of variability (CV) was associated with a significant decrease of HDL cholesterol. HbA1c and CGM were not associated with total cholesterol or LDL cholesterol.

**Conclusions:**

We observed a negative association between CV and HDL cholesterol levels and a positive association between hyperglycemia metrics and triglyceride levels. These findings suggest that CGM parameters may be a helpful tool to guide the improvement of both glycemic control and lipid profile in T1D.

## Introduction

Patients with Type 1 diabetes (T1D) have an increased risk of cardiovascular disease in comparison with the general population. This is observed both in those who have very poor glycemic control (8 to 10 times as high) and in those who have a glycated hemoglobin level of 6.9% or lower (twice as high) [1]. One of the contributing factors is the elevated concentration of triglycerides and low density lipoproteins (LDL) cholesterol, even after adjustment for age and glycated hemoglobin (ΗbΑ1c) levels [2].

T1D with poor glycemic control is associated with quantitative abnormalities of lipoproteins, such as increased triglycerides and LDL cholesterol. Contrastingly, comparing with the general population, T1D with optimal glycemic control have normal or slightly decreased triglycerides and LDL cholesterol, whereas high density lipoproteins (HDL) cholesterol is normal or slightly increased [3]. Hence, poor glycemic control can lead to abnormal lipid levels and atherogenic changes in lipoprotein composition [4]. However, it is important to highlight that even in patients with good glycemic control and no additional cardiovascular risk factors there is a significantly increased risk of cardiovascular events. This suggests that factors other than chronic hyperglycemia and traditional cardiovascular risk factors might be involved [3].

In the Coronary Artery Calcification in Type 1 Diabetes (CACTI) study, LDL cholesterol, non-HDL cholesterol, and triglycerides levels rose 4.0 mg/dL, 5.0 mg/dL and 4.6 mg/dL, respectively, as HbA1c increased 1% [5]. So far, the classic method for assessing glycemic control has been the measurement of HbA1c [6]. Nonetheless, this method has several limitations, such as not reflecting intra- and inter-day glycemic fluctuations [6]. Significant glycemic excursions, including hypoglycemia and postprandial hyperglycemia, have been associated with microvascular and macrovascular complications, although most previous studies only evaluated patients with Type 2 Diabetes (T2D) [7, 8]. Whether these glycemic excursions are associated with changes in lipid profile is uncertain [9].

Continuous glucose monitoring (CGM) addresses many of the limitations inherent to HbA1c evaluation [6]. Percentages of time in range such as time in target range (TIR), time below range (TBR), and time above range (TAR) should be reported as key diabetes control metrics in clinical practice and in clinical studies [6]. The most recent glycemic targets incorporate both parameters, HbA1c and CGM. Thus, the American Diabetes Association (ADA) suggests glycemic goals should be an HbA1C goal of <7% (53 mmol/mol) and, if using a continuous glucose monitoring device, a TIR of > 70% with TBR < 4% [10].

CGM also allows the evaluation of the glycemic variability. The glycemic variability is characterized by the amplitude, frequency, and duration of the fluctuation [6]. Glycemic variability may also be a clinically valuable marker of T1D management, expanding the understanding of glycemic control beyond HbA1c alone and time in range [6]. Numerous studies have underlined glycemic variability as an independent risk factor for diabetes complications, particularly cardiovascular disease [8, 11–13] and its impact on cognitive function and quality of life [14]. However, the effect of the glycemic variability and time in range on the lipid profile in T1D has not yet been investigated in detail.

In the present study, we aimed to analyze the association of glycemic control with lipid profile in T1D, focusing on the assessment of glycemic variability and time in range obtained from CGM.

## Materials and methods

We performed a retrospective observational cohort, collecting data from the medical records of T1D patients followed in the Endocrinology Department of *Centro Hospitalar Universitário de São João* (CHUSJ) in Porto (Portugal) between 1^st^ of January of 2019 and 30^th^ of November of 2022. This study was approved by the ethics committee of CHUSJ.

We included patients with T1D aged 18 years or older using a Freestyle Libre CGM with data uploaded in the Libreview plataform. Exclusion criteria were lack of available data (patients missing one of these parameters were excluded: lipid profile or data from the CGM) and having a percentage of active CGM time <70%, following the International Consensus on the Use of the Glucose Flash Monitoring System [6]. The study was divided into two phases: A) Cross-sectional evaluation of the association of CGM data with lipid profile and B) Longitudinal evaluation of the association of variations of CGM data with variations of lipid profile (6 to 18 months of interval between evaluations). For each moment, the date for collection of CGM data was matched with the date of lipid profile evaluation (less than 3 months apart). Participants with data available from only one timepoint were included only in the cross-section evaluation, and participants with at least two timepoints available were included in the cross-sectional evaluation and in the longitudinal evaluation.

Clinical characteristics collected at baseline include sex, age, educational level, profession, duration of diabetes, physical activity, smoking and drinking habits, family history of T1D and T2D, hypertension, dyslipidemia, and complications of T1D. Physical activity was graded as 0 (no activity or less than once a week) or 1 (twice or more a week). Smoking was defined as never smoked or smokes regularly. Alcohol intake was defined as no consumption/moderate consumption (women that drink less than one drink a day or men that drink less than two drinks a day) and excessive consumption (women that drink more than one drink a day or men that drink more than two drinks a day). Hypertension was defined as systolic blood pressure (SBP) ≥ 140 mm Hg and/or diastolic blood pressure (DBP) ≥ 90 mm Hg in ≥ 2 different appointments or treatment with anti-hypertensive drugs. Atherosclerotic cardiovascular disease (ASCVD) was defined as having ischemic heart disease, history of arterial revascularization, stroke, or TIA and/or peripheral artery disease. Heart failure was defined as having B-type natriuretic peptide (BNP) ≥ 35 pg/mL and structural and/or functional echocardiographic changes. Nephropathy was defined as having persistent (≥ 3 months) albuminuria (≥ 30 mg/day) and/or reduced eGFR. In addition, for each timepoint, we also collected data regarding insulin pump use, type of insulin, total daily dose (TDD), use of lipid lowering drugs, use of other medication, height, weight, and body mass index (calculated as weight/height^2^), waist circumference, total cholesterol, HDL cholesterol, LDL cholesterol, triglycerides, plasma creatinine, HbA1C and albuminuria. The following CGM parameters were collected in each timepoint: time in range (TIR, 70–180 mg/dL), time below range (TBR, < 70 mg/dL, and TB54, ≤ 54 mg/dL), time above range (TAR, ≥ 181 mg/dL, and TA250, ≥ 250 mg/dL), glycemic variability (coefficient of variability, CV) and glucose management indicator (GMI).

### Statistical analysis

The cross-sectional associations of categories of TIR ( < 50%, ≥ 50 to < 70% and ≥ 70%) and CV ( ≤ 36%, > 36 to < 45% and ≥ 45%) with lipid profile were evaluated with ANOVA and the Kruskall-Wallis test for normal and non-normal continuous variables, respectively. The association of CGM parameters and HbA1c with lipid profile was evaluated with linear regression models unadjusted and adjusted for sex and age. Non-normal variables (triglycerides, TAR, TA250, TBR and TB54) were log-transformed for inclusion in the linear regression models. The association of variations of CGM parameters with HbA1c with variations of lipid profile was evaluated with unadjusted and adjusted for sex and age linear regression models. Participants that changed statin treatment during the follow-up period were not included in the longitudinal analysis. Continuous variables are described as mean ± standard deviation for normally distributed variables or median (25th–75th percentiles) for variables with non-normal distribution. Categorical variables are presented as number and percentages. For each individual analysis, participants with missing values were not included. All analyses were conducted with the statistical software package Stata IC version 17.0. A two-sided *P*-value < 0.05 was considered statistically significant.

## Results

### Baseline characteristics of the study population

Five hundred and thirty-five T1D patients using a CGM were followed in the Endocrinology Department of CHUSJ during the study period. Of these, we excluded 61 due to lack on available data and 232 due to having a percentage of active CGM time < 70%. The final number of individuals analyzed was 242 (Supplementary Fig. 1).

The baseline characteristics of the study population are shown in Table 1a, b. Among the cohort of 242 patients, 51.7% were men, the mean age (± standard deviation) was 36.6 ± 12.6 years and 56.7% had higher education. Concerning the T1D related parameters (Table 1a), the mean duration of this disease was 16.8 ± 10.3 years and 35.1% of the patients had an insulin pump. The mean baseline TIR was 56.7 ± 18.2% and the mean baseline CV was 38.5 ± 7.7%. The mean TDD was 52.0 ± 21.7 UI and the mean TDD/kg was 0.7 ± 0.3 UI/kg. Regarding the dyslipidemia related parameters (Table 1b), 69.8% of the patients were not treated with a statin and, of those who were, 5.5%, 45.2% and 49.3%, were treated, respectively, with a low, moderate, and high potency statin. None of these patients was under fibrates. The mean baseline HDL cholesterol level was 57.3 ± 14.5 mg/dL, and the mean triglyceride level was 84.1 ± 78.1 mg/dL.

**Table 1:** Baseline characteristics of the study population (*n* = 242). **a:** Baseline characteristics of the study population such as demographic and social features and T1D monitoring values. Missing data for each variable shown in the supplementary Table 1A. **b**: Baseline characteristics of the study population such as statin treatment and lipid profile analysis. Missing data for each variable shown in the supplementary Table 1B

a	
Male sex, *n* (%)	124 (51.7%)
Age, years	36.6 ± 12.6
Educational level, *n* (%)	
Less than 9th grade	24 (11.2%)
9th to 12th grade	69 (32.1%)
Higher Education	122 (56.7%)
Duration of diabetes, years	16.8 ± 10.3
With insulin pump, *n* (%)	85 (35.1%)
Body Mass Index, kg/m^2^	24.8 ± 3.9
Physical activity, *n* (%)	92 (46.2%)
With smoking habits, *n* (%)	43 (23.8%)
With drinking habits, *n* (%)	22 (12.4%)
Hypertension, *n* (%)	32 (13.2%)
ASCVD, *n* (%)	12 (5.0%)
Nephropathy, *n* (%)	21 (8.7%)
Retinopathy, *n* (%)	53 (22.2%)
Neuropathy, *n* (%)	10 (4.1%)
Heart failure, *n* (%)	5 (2.1%)
HbA1C, %	7.6 ± 1.3
GMI, %	7.4 ± 0.9
Time in range, %	56.7 ± 18.2
Time below range, %	6.6 ± 8.2
Time below 54 mg/dL, %	2.3 ± 4.2
Time above range, %	42.9 ± 26.7
Time above 250 mg/dL, %	15.4 ± 15.0
CV, %	38.5 ± 7.7
Waist circunference, cm	84.9 ± 13.2
TDD, IU	52.0 ± 21.7
TDD/kg, IU/kg	0.7 ± 0.3
**b**	
Without statin, *n* (%)	169 (69.8%)
With statin, *n* (%)	73 (30.2%)
Atorvastatin, *n* (%)	50 (20.7%)
Rosuvastatin, *n* (%)	19 (7.9%)
Simvastatin, *n* (%)	4 (1.7%)
Statin Potency	
Low, *n* (%)	4 (5.5%)
Moderate, *n* (%)	33 (45.2%)
High, *n* (%)	36 (49.3%)
With Ezetimibe	12 (5.0%)
Total cholesterol, mg/dL	166.5 ± 37.2
HDL cholesterol, mg/dL	57.3 ± 14.5
LDL cholesterol, mg/dL	95.0 ± 29.2
Triglycerides, mg/dL	84.1 ± 78.1
Non-HDL cholesterol, mg/dL	109.5 ± 31.6

*ASCVD* Atherosclerotic Cardiovascular Disease, *HbA1C* Hemoglobin A1C, *GMI* Glucose management indicator, *CV* Coefficient of variability, *TDD* Total Daily Dose, *LDL* Low-density lipoprotein, *HDL* High-density lipoprotein.

When comparing baseline characteristics of participants with and without an insulin pump (supplemental Table 3), even though the first group had lower levels of HbA1C (*p* = 0.020), there were no statistically significant differences found in CGM parameters and lipid profile analysis. Moreover, when comparing baseline characteristics of participants with normal BMI and obesity (supplemental Table 4), the second group had lower levels of HDL cholesterol (*p* = 0.014) and higher levels of triglycerides (*p* = 0.001) as it would be expected.

### Cross-sectional evaluation

In the cross-sectional evaluation (*n* = 242), higher TIR levels were associated with lower triglyceride levels (Table 2a). The mean triglyceride levels were 75.5 [57.0, 109.0] mg/dL in patients with TIR < 50%, 65.5 [51.0, 90.0] mg/dL in patients with TIR between 50 and 70% and 64.0 [51.0, 80.0] mg/dL in patients with TIR ≥ 70% (*p* = 0.006). TIR levels were not significantly associated with total cholesterol, HDL cholesterol, LDL cholesterol or non-HDL cholesterol levels.

**Table 2:** Cross-sectional associations of categories of TIR (Table 1a) and CV (Table 1b) with lipid profile. **a**: Cross-sectional associations of categories of TIR ( < 50%, ≥ 50 to < 70% and ≥ 70%) with total cholesterol, HDL cholesterol, LDL cholesterol, triglycerides and non-HDL cholesterol. **b**: Cross-sectional associations of categories of CV ( ≤ 36%, > 36 to < 45% and ≥45%) with total cholesterol, HDL cholesterol, LDL cholesterol, triglycerides and non-HDL cholesterol

a	TIR < 50%, *n* = 86	TIR ≤ 50% and > 70%, *n* = 103	TIR ≥ 70%, *n* = 53	*P*-value
Total cholesterol, mg/dL	166.1 ± 38.3	165 ± 37.0	169.5 ± 36.5	0.66
HDL cholesterol, mg/dL	56.8 ± 15.1	56.0 ± 14.2	60.6 ± 14.1	0.60
LDL cholesterol, mg/dL	93.8 ± 29.7	94.5 ± 26.0	97.6 ± 33.8	0.50
Triglycerides, mg/dL	**75.5 [57.0, 109.0]**	**65.5 [51.0, 90.0]**	**64.0 [51.0, 80.0]**	**0.006**
Non-HDL cholesterol, mg/dL	109.5 ± 32.2	109.7 ± 30.0	109.1 ± 34.0	0.95

b	CV ≤ 36%, *n* = 90	CV < 36% and > 45%, *n* = 104	CV ≥ 45%, *n* = 48	*P*-value
Total cholesterol, mg/dL	170.1 ± 34.3	166.7 ± 36.7	159.2 ± 43.3	0.12
HDL cholesterol, mg/dL	**58.4** ± **14.6**	**58.8** ± **15.2**	**51.8** ± **11.6**	**0.035**
LDL cholesterol, mg/dL	98.9 ± 29.8	91.4 ± 27.3	94.7 ± 31.3	0.30
Triglycerides, mg/dL	65.0 [53.0, 87.0]	66.0 [51.0, 98.0]	73.0 [59.0, 105.0]	0.08
Non-HDL cholesterol, mg/dL	112.5 ± 30.5	107.9 ± 30.6	107.4 ± 35.8	0.33

Parameters with a significant difference (*p* < 0.05) are highlighted in bold.

*TIR* Time in range, *LDL* Low-density lipoprotein, *HDL* High-density lipoprotein, *CV* Coefficient of Variability.

Regarding the association of CV with lipid profile in the cross-sectional analysis, lower CV levels were associated with higher HDL cholesterol levels. As CV decreases, HDL levels are higher: 58.4 ± 14.6 mg/dL in patients with CV ≤ 36%, 58.8 ± 15.2 mg/dL in patients with CV between 36 and 45% and 51.8 ± 11.6 mg/dL in patients with CV > 45% (*p* = 0.035). Total cholesterol, LDL cholesterol, triglycerides and non-HDL cholesterol are not significantly associated with CV levels (Table 2b).

In Table 3, we present the unadjusted and adjusted results of the linear regressions of the association of HbA1c and CGM parameters with lipid profile. Higher TIR levels were associated with lower triglycerides levels in the unadjusted and adjusted analysis. Higher HbA1C, GMI, TAR and TA250 were also associated with higher triglycerides levels in both analyses. The only glycemic parameter associated with HDL cholesterol was CV (−0.30 mg/dL, 95%CI −0.54 to −0.06, per 1% increase in CV in the adjusted analysis, *p* = 0.015). Neither HbA1c nor CGM parameters were significantly associated with total cholesterol, LDL cholesterol and non-HDL cholesterol.

**Table 3:** Cross-sectional evaluation (*n* = 242)

	HbA1c (%)		GMI (%)		TIR (%)		TBR (%)	
	β (95% CI)	*P*-Value	β (95% CI)	P Value	β (95% CI)	*P*-Value	β (95% CI)	*P*-Value
**Total Cholesterol (mg/dL)**								
Unadjusted	1.62 (−2.25 to 5.49)	0.41	1.14 (−4.26 to 6.54)	0.68	−0.08 (−0.35 to 0.20)	0.58	−0.37 (−5.80 to 5.05)	0.89
Adjusted	2.35 (−1.48 to 6.17)	0.23	2.12 (−3.24 to 7.48)	0.44	−0.14 (−0.41 to 0.13)	0.31	−0.33 (−5.72 to 5.06)	0.90
**HDL Cholesterol (mg/dL)**								
Unadjusted	−0.35 (−1.84 to 1.14)	0.64	−0.44 (−2.53 to 1.65)	0.68	0.04 (−0.06 to 0.15)	0.42	0.16 (−1.93 to 2.24)	0.88
Adjusted	−0.48 (−1.95 to 0.98)	0.52	−0.47 (−2.52 to 1.57)	0.65	0.03 (−0.07 to 0.14)	0.53	0.15 (−1.91 to 2.20)	0.89
**LDL Cholesterol (mg/dL)**								
Unadjusted	0.50 (−2.66 to 3.65)	0.76	0.91 (−3.61 to 5.43)	0.70	−0.04 (−0.26 to 0.17)	0.70	−0.53 (−4.88 to 3.82)	0.81
Adjusted	1.15 (−1.95 to 4.26)	0.47	1.75 (−2.71 to 6.20)	0.44	−0.08 (−0.30 to 0.13)	0.45	−0.40 (−4.69 to 3.88)	0.85
**Triglycerides (mg/dL)**								
Unadjusted	**0.12 (0.07 to 0.16)**	**0.000**	**0.10 (0.03 to 0.16)**	**0.004**	**−0.01 (−0.01 to −0.00)**	**0.003**	−0.02 (−0.09 to 0.05)	0.64
Adjusted	**0.12 (0.07 to 0.17)**	**0.000**	**0.09 (0.03 to 0.16)**	**0.006**	**−0.00 (−0.01 to −0.00)**	**0.005**	−0.01 (−0.08 to 0.06)	0.69
**Non HDL Cholesterol (mg/dL)**								
Unadjusted	2.03 (−1.24 to 5.31)	0.22	1.68 (−2.90 to 6.26)	0.47	−0.12 (−0.35 to 0.11)	0.31	−0.77 (−5.44 to 3.90)	0.75
Adjusted	2.85 (−0.39 to 6.09)	0.085	2.63 (−1.91 to 7.17)	0.26	−0.17 (−0.40 to 0.06)	0.15	−0.68 (−5.29 to 3.94)	0.77

	TB54 (%)		TAR (%)		TA250 (%)		CV (%)	
	β (95% CI)	*P*-Value	β (95% CI)	*P*-Value	β (95% CI)	*P*-Value	β (95% CI)	*P*-Value
**Total Cholesterol (mg/dL)**								
Unadjusted	−**7.75 (−14.89 to −0.62)**	**0.033**	−0.06 (−6.81 to 6.69)	0.99	0.09 (−4.77 to 4.96)	0.97	−0.46 (−1.10 to 0.19)	0.17
Adjusted	−6.78 (−13.93 to 0.36)	0.063	1.74 (−5.00 to 8.49)	0.61	1.17 (−3.67 to 6.01)	0.63	−0.35 (−0.99 to 0.30)	0.29
**HDL Cholesterol (mg/dL)**								
Unadjusted	−2.42 (−5.39 to 0.55)	0.11	−1.78 (−4.38 to 0.81)	0.18	−0.19 (−2.02 to 1.64)	0.84	**−0.33 (−0.58 to −0.09)**	**0.008**
Adjusted	−1.69 (−4.65 to 1.28)	0.26	−1.42 (−3.97 to 1.14)	0.28	0.06 (−1.73 to 1.84)	0.95	**−0.30 (−0.54 to −0.06)**	**0.015**
**LDL Cholesterol (mg/dL)**								
Unadjusted	−5.25 (−10.70 to 0.19)	0.058	1.12 (−4.31 to 6.55)	0.68	0.09 (−3.75 to 3.94)	0.96	−0.25 (−0.77 to 0.26)	0.33
Adjusted	−4.59 (−10.04 to 0.86)	0.098	2.46 (−2.91 to 7.84)	0.37	0.85 (−2.95 to 4.65)	0.66	−0.19 (−0.70 to 0.31)	0.46
**Triglycerides (mg/dL)**								
Unadjusted	−0.02 (−0.10 to 0.07)	0.71	**0.12 (0.03 to 0.20)**	**0.006**	**0.09 (0.03 to 0.14)**	**0.005**	0.01 (−0.00 to 0.02)	0.084
Adjusted	−0.02 (−0.11 to 0.07)	0.68	**0.11 (0.02 to 0.19)**	**0.013**	**0.08 (0.02 to 0.14)**	**0.008**	0.01 (−0.00 to 0.01)	0.12
**Non HDL Cholesterol (mg/dL)**								
Unadjusted	−5.33 (−11.30 to 0.63)	0.079	1.88 (−3.84 to 7.60)	0.52	0.38 (−3.73 to 4.49)	0.86	−0.17 (−0.72 to 0.38)	0.54
Adjusted	−5.10 (−11.06 to 0.87)	0.093	3.28 (−2.42 to 8.99)	0.26	1.19 (−2.89 to 5.28)	0.57	−0.09 (−0.64 to 0.45)	0.74

Cross-sectional evaluation of the association of CGM data with lipid profile. Triglycerides, TBR, TB54, TAR and TA250 values were log-transformed for the regression. Statistically significant differences (*p* < 0.05) are highlighted in bold.

*Adjusted model* adjusted for age and sex, *LDL* Low-density lipoprotein, *HDL* High-density lipoprotein, *HbA1C* Hemoglobin A1C, *GMI* Glucose management indicator, *TIR* Time in range, *TBR* Time below range (< 70 mg/dL), *TB54* Time below 54 mg/dL, *TAR* Time above range (≥ 181 mg/dL), *TA250* Time above 250 mg/dL), *CV* Coefficient of variability, *CGM* Continuous Glucose Monitoring.

### Longitudinal evaluation

In the longitudinal evaluation, we included the 90 participants with two different moments of analysis (6 to 18 months apart) (characteristics of these participants are shown in supplementary Table 2A, B). Of these participants, 23 were treated with statins and 17 maintained the same statin treatment between periods. The time between evaluations was 10.5 ± 3.0 months. The mean variation of TIR was 0.1 ± 13.4%, of CV was −1.0 ± 6.8 % and of HbA1c was −0.1 ± 0.7 %. As shown in Table 4, there was a significant association between the variation of CV and the variation of HDL cholesterol levels, both in the unadjusted and adjusted models (−0.54 mg/dL, 95%CI (−0.95 to −0.13), per 1% increase in CV in the adjusted analysis, p = 0.010). The variation of TBR was significantly associated with the variation of HDL cholesterol in both models (−0.46 mg/dL, 95%CI (−0.91 to −0.00), per 1% increase in TBR in the adjusted analysis, *p* = 0.048). Similarly to the previous analysis, neither HbA1c nor CGM parameters were significantly associated with total cholesterol, LDL cholesterol and non-HDL cholesterol. Differently from the cross-sectional analysis, variation of TIR, HbA1C, GMI, TAR and TA250 were not significantly associated with the variation of triglycerides values.

**Table 4:** Longitudinal evaluation (n = 90)

	HbA1c (%)		GMI (%)		TIR (%)		TBR (%)	
	β (95% CI)	*P*-Value	β (95% CI)	*P*-Value	β (95% CI)	*P*-Value	β (95% CI)	*P*-Value
**Total Cholesterol (mg/dL)**								
Unadjusted	−3.61 (−13.82 to 6.60)	0.48	−3.44 (−11.89 to 5.02)	0.42	0.29 (−0.14 to 0.72)	0.17	−0.28 (−1.21 to 0.65)	0.56
Adjusted	−4.05 (−14.65 to 6.56)	0.45	−3.67 (−12.31 to 4.96)	0.40	0.30 (−0.13 to 0.74)	0.17	−0.27 (−1.22 to 0.67)	0.57
**HDL Cholesterol (mg/dL)**								
Unadjusted	1.46 (−3.55 to 6.47)	0.56	1.84 (−2.34 to 6.02)	0.38	−0.04 (−0.25 to 0.18)	0.73	**−0.45 (−0.90 to −0.00)**	**0.049**
Adjusted	1.94 (−3.21 to 7.09)	0.45	1.96 (−2.28 to 6.21)	0.36	−0.04 (−0.26 to 0.18)	0.71	**−0.46 (−0.91 to −0.00)**	**0.048**
**LDL Cholesterol (mg/dL)**								
Unadjusted	−2.96 (−11.65 to 5.73)	0.50	−5.22 (−13.07 to 2.63)	0.19	0.30 (−0.10 to 0.70)	0.14	0.20 (−0.77 to 1.16)	0.69
Adjusted	−2.74 (−11.72 to 6.25)	0.55	−5.10 (−13.10 to 2.91)	0.21	0.29 (−0.12 to 0.70)	0.16	0.19 (−0.79 to 1.17)	0.70
**Triglycerides (mg/dL)**								
Unadjusted	4.50 (−7.08 to 16.07)	0.44	0.62 (−9.39 to 10.63)	0.90	0.04 (−0.47 to 0.55)	0.88	−0.25 (−1.35 to 0.85)	0.65
Adjusted	3.99 (−7.93 to 15.91)	0.51	0.18 (−9.94 to 10.29)	0.97	0.06 (−0.46 to 0.58)	0.82	−0.23 (−1.33 to 0.88)	0.68
**Non-HDL Cholesterol (mg/dL)**								
Unadjusted	−5.25 (−14.29 to 3.80)	0.25	−5.37 (−12.80 to 2.06)	0.15	0.33 (−0.05 to 0.71)	0.09	0.17 (−0.65 to 1.00)	0.68
Adjusted	−6.20 (−15.53 to 3.12)	0.19	−5.74 (−13.31 to 1.82)	0.13	0.35 (−0.04 to 0.73)	0.08	0.19 (−0.65 to 1.02)	0.66

	TB54 (%)		TAR (%)		TA25 (%)		VC (%)	
	β (95% CI)	*P*-Value	β (95% CI)	*P*-Value	β (95% CI)	*P*-Value	β (95% CI)	*P*-Value
**Total Cholesterol (mg/dL)**								
Unadjusted	−0.37 (−2.04 to 1.29)	0.66	−0.18 (−0.47 to 0.11)	0.22	−0.09 (−0.50 to 0.32)	0.67	−0.66 (−1.50 to 0.19)	0.13
Adjusted	−0.36 (−2.06 to 1.33)	0.67	−0.19 (−0.49 to 0.10)	0.20	−0.10 (−0.53 to 0.32)	0.63	−0.66 (−1.51 to 0.20)	0.13
**HDL Cholesterol (mg/dL)**								
Unadjusted	−0.54 (−1.36 to 0.28)	0.19	0.00 (−0.14 to 0.15)	0.95	0.01 (−0.20 to 0.21)	0.94	**−0.55 (−0.96 to −0.15)**	**0.008**
Adjusted	−0.55 (−1.37 to 0.28)	0.19	0.00 (−0.14 to 0.15)	0.97	0.01 (−0.20 to 0.22)	0.91	−**0.54 (−0.95 to −0.13)**	**0.010**
**LDL Cholesterol (mg/dL)**								
Unadjusted	0.17 (**−**1.70 to 2.04)	0.86	**−**0.14 (**−**0.41 to 0.13)	0.30	**−**0.00 (**−**0.39 to 0.39)	0.99	**−**0.08 (**−**0.92 to 0.75)	0.84
Adjusted	0.16 (**−**1.76 to 2.07)	0.87	**−**0.14 (**−**0.41 to 0.14)	0.33	0.01 (**−**0.39 to 0.41)	0.96	**−**0.09 (**−**0.94 to 0.76)	0.84
**Triglycerides (mg/dL)**								
Unadjusted	**−**0.20 (**−**2.17 to 1.77)	0.84	**−**0.02 (**−**0.36 to 0.33)	0.92	**−**0.06 (**−**0.55 to 0.42)	0.80	**−**0.64 (**−**1.64 to 0.36)	0.21
Adjusted	**−**0.17 (**−**2.15 to 1.80)	0.86	**−**0.02 (**−**0.37 to 0.33)	0.91	**−**0.09 (**−**0.58 to 0.41)	0.72	**−**0.70 (**−**1.70 to 0.31)	0.17
**Non-HDL Cholesterol (mg/dL)**								
Unadjusted	0.15 (**−**1.33 to 1.63)	0.84	**−**0.18 (**−**0.44 to 0.07)	0.15	**−**0.09 (**−**0.46 to 0.27)	0.61	**−**0.10 (**−**0.86 to 0.66)	0.79
Adjusted	0.17 (**−**1.33 to 1.67)	0.82	**−**0.20 (**−**0.46 to 0.06)	0.13	**−**0.11 (**−**0.49 to 0.26)	0.55	**−**0.11 (**−**0.88 to 0.66)	0.78

Longitudinal evaluation of the association of variations of CGM data with variations of lipid profile (6 to 18 months of interval between evaluations). Statistically significant differences (*p* < 0.05) are highlighted in bold.

*Adjusted model* adjusted for age and sex, *LDL* Low-density lipoprotein, *HDL* High-density lipoprotein, *HbA1C* Hemoglobin A1C, *GMI* Glucose management indicator, *TIR* Time in range, *TBR* Time below range (< 70 mg/dL), *TB54* Time below 54 mg/dL, *TAR* Time above range (≥ 181 mg/dL), *TA250* Time above 250 mg/dL), *CV* Coefficient of variability, *CGM* Continuous Glucose Monitoring.

## Discussion

In this study of the association of glycemic control with lipid profile in T1D, the most prominent finding was the negative relationship between CV and HDL cholesterol levels. We also found that HbA1C, TIR and CV levels are significantly negatively correlated with triglycerides levels, even after adjustments. Of note, CGM parameters were not significantly associated with total cholesterol and LDL cholesterol.

An improvement in the HDL cholesterol values as CV decreases was observed in the cross-sectional and in the longitudinal analyses. Indeed, T1D patients frequently have reduced levels of HDL-cholesterol, particularly when they have poor glycemic control [15]. Moreover, in the longitudinal analysis, increases in TBR levels were significantly correlated with decreases of HDL cholesterol levels. This is probably related to the significant association of increased glycemic variability with hypoglycemia [6]. However, the mechanisms for this decrease in HDL are still unsettled.

Higher levels of TIR were significantly associated with triglycerides values in the cross-sectional evaluation. In addition, a positive correlation of triglyceride levels was also found with HbA1C, GMI, TAR and TA250, suggesting that hyperglycemia leads to hypertriglyceridemia. This is probably a consequence of reduced lipoprotein lipase (LPL) activity secondary to insulin deficiency in T1D [17]. Contrastingly, in well-controlled patients, triglyceride levels are usually normal or even lower than the general population due to peripheral hyperinsulinemia [17]. The administration of subcutaneous insulin increases LPL activity and subsequently very low density lipoproteins (VLDL) turnover, resulting in the so called “supernormal” lipid profile seen in T1D patients [18].

In former studies, T1D patients with poor glycemic control frequently have higher LDL cholesterol levels, whereas those with optimal glycemic control have LDL cholesterol levels that are normal or even slightly decreased [3]. This is also part of the abovementioned “supernormal” lipid profile [18]. The increased LDL levels in T1D patients with poor glycemic control may be related not only to high plasma glucose levels but also to other factors (e.g., diet and physical activity). LDL cholesterol levels may not increase as a direct consequence of poor glycemic control in T1D, as both production and catabolism of LDL are increased with hyperglycemia [19]. Our results support the hypothesis that hyperglycemia and glycemic variability have more influence on HDL cholesterol and triglycerides that on LDL cholesterol.

Dyslipidemia is likely to be one of main reasons for the accelerated macrovascular disease in T1D [19]. Patients with T1D and cardiovascular disease have lower levels of HDL cholesterol and higher triglycerides levels than those without cardiovascular disease [20]. Furthermore, triglycerides and LDL cholesterol levels are associated with cardiovascular events even in patients with lipid profile within "normal range” [2]. Therefore, treatment of lipid abnormalities has the potential to markedly reduce cardiovascular events [19]. Likewise, ADA recommends an intensification of the lifestyle therapy and optimization of glycemic control for patients with elevated triglycerides levels (≥150 mg/dL) and/or low HDL cholesterol (<40 mg/dL for men, <50 mg/dL for women) [21]. Our study suggests that these lipid abnormalities can be corrected through monitoring and improvement of TIR and CV, which further reinforces the role of CGM systems in addition to the traditional blood glucose determinations. When properly used, these systems can lead to better glucose control, and consequently to an improvement in the lipid profile, through more personalized and rigorous clinical advice [22].

Our study has several limitations. The main limitation is its retrospective study design based on clinical records. This limits the evaluation to routinely assessed variables in clinical practice. Some variables such as physical activity, smoking and alcohol intake could also be informative, yet they were not available for every participant. Our study was conducted in a single center and only in adults, which limits the generalizability of our results. Finally, the significant association between triglycerides and TIR levels was only observed in the cross-sectional evaluation, but not in the longitudinal evaluation. This may be because most patients had small variations of triglycerides and TIR levels in the longitudinal analysis which may have decreased the power of our analysis to detect significant associations.

## Conclusion

In conclusion, we observed a negative association between CV and HDL cholesterol and a positive association between hyperglycemia (as assessed by HbA1C, TIR and TAR) and triglyceride levels. The beneficial effects of increasing TIR and decreasing CV on triglycerides and HDL cholesterol levels, respectively, suggest that CGM may be a helpful tool to guide the improvement of both glycemic control and lipid profile in T1D. Further studies such as randomized clinical trials are needed to better characterize the effects of the improvement of CGM parameters on plasma lipid profile and on the risk of atherosclerotic cardiovascular disease in T1D.

### Supplementary information

Supplemental Figure 1

Supplemental table 1

Supplemental table 2

Supplemental table 3

Supplemental table 4
