# Supplementary material for: Association of glycemic variability and time in range with lipid profile in type 1 diabetes
Source: Endocrine. 2023 Dec 5;83(1):69–76. doi: 10.1007/s12020-023-03464-x (PMC10805887; doi:10.1007/s12020-023-03464-x)
Supplement: Supplementary file 1 — Supplemental Figure 1 [file 12020_2023_3464_MOESM1_ESM.docx]

**Supplemental Figure 1**

**535** adult patients with T1D using a CGM followed in CHUSJ

61 patients did not have available data:

lipid profile and/or CGM data

**474** adult patients with T1D using a CGM with available data

232 patients with a percentage of active CGM time < 70%

**242** adult patients with T1D using a CGM with a percentage of active CGM time ≥ 70% included in this study

**Supplemental Figure 1 caption:**

Flow diagram illustrating the selection of the study sample.

T1D: Type 1 diabetes; CHUSJ: Centro Hospitalar Universitário de São João; CGM: continuous glucose monitoring.
