# Supplementary material for: Association of glycemic variability and time in range with lipid profile in type 1 diabetes
Source: Endocrine. 2023 Dec 5;83(1):69–76. doi: 10.1007/s12020-023-03464-x (PMC10805887; doi:10.1007/s12020-023-03464-x)
Supplement: Supplementary file 2 — Supplemental table 1 [file 12020_2023_3464_MOESM2_ESM.docx]

**Supplemental table 1**

| Supplemental table 1a | n | % |
| --- | --- | --- |
| Male sex | 2 | 0.83 |
| Age | 0 | 0 |
| Educational level | 27 | 11.16 |
| Duration of diabetes | 9 | 3.72 |
| With insulin pump | 0 | 0 |
| Body Mass Index | 45 | 18.6 |
| Physical activity | 43 | 17.77 |
| With smoking habits | 61 | 25.21 |
| With drinking habits | 65 | 26.86 |
| Hypertension | 0 | 0 |
| ASCVD | 0 | 0 |
| Nephropathy | 0 | 0 |
| Retinopathy | 3 | 1.24 |
| Neuropathy | 1 | 0.41 |
| Heart failure | 2 | 0.83 |
| HbA1C | 12 | 4.96 |
| GMI | 0 | 0 |
| Time in range | 0 | 0 |
| Time below range | 0 | 0 |
| Time below 54 mg/dL | 0 | 0 |
| Time above range | 0 | 0 |
| Time above 250 mg/dL | 0 | 0 |
| CV | 0 | 0 |
| Waist circumference | 211 | 87.19 |
| TDD | 161 | 66.53 |
| TDD/kg | 169 | 69.83 |

| Supplemental table 1b | n | % |
| --- | --- | --- |
| Statin | 0 | 0 |
| Statin Potency | 0 | 0 |
| Ezetimibe | 0 | 0 |
| Total cholesterol | 15 | 6.2 |
| HDL cholesterol | 13 | 5.37 |
| LDL cholesterol | 32 | 13.22 |
| Triglycerides | 9 | 3.72 |
| Non-HDL cholesterol | 16 | 6.61 |

**Supplemental table 1 caption:**

Missing data of the baseline characteristics of the study population (n=242).

**Supplemental table 1a:** Missing data of the baseline characteristics of the study population such as demographic and social features and T1D monitoring values.

**Supplemental table 1b:** Missing data of the baseline characteristics of the study population such as statin treatment and lipid profile analysis.

ASCVD: Atherosclerotic Cardiovascular Disease; HbA1C: Hemoglobin A1C; GMI: Glucose management indicator; CV: Coefficient of variability; TDD: Total Daily Dose; LDL: Low-density lipoprotein; HDL: High-density lipoprotein.
