# Supplementary material for: Association of glycemic variability and time in range with lipid profile in type 1 diabetes
Source: Endocrine. 2023 Dec 5;83(1):69–76. doi: 10.1007/s12020-023-03464-x (PMC10805887; doi:10.1007/s12020-023-03464-x)
Supplement: Supplementary file 3 — Supplemental table 2 [file 12020_2023_3464_MOESM3_ESM.docx]

**Supplemental table 2**

| Supplemental table 2a |  |
| --- | --- |
| Male sex, n (%) | 50 (55.6%) |
| Age, years | 35.2 ± 11.6 |
| Educational level, n (%) |  |
| Less than 9^th^ grade | 5 (6.2%) |
| 9^th^ to 12^th^ grade | 23 (28.4%) |
| Higher Education | 53 (65.4%) |
| Duration of diabetes, years | 17.4 ± 9.7 |
| With insulin pump, n (%) | 44 (48.9%) |
| Body Mass Index, kg/m^2^ | 24.8 ± 3.9 |
| Physical activity, n (%) | 42 (55.3%) |
| With smoking habits, n (%) | 10 (15.9%) |
| With drinking habits, n (%) | 3 (4.8%) |
| Hypertension, n (%) | 10 (11.1%) |
| ASCVD, n (%) | 4 (4.4%) |
| Nephropathy, n (%) | 10 (11.1%) |
| Retinopathy, n (%) | 18 (20.0%) |
| Neuropathy, n (%) | 4 (4.4%) |
| Heart failure, n (%) | 0 |
| HbA1C, % | 7.4 ± 1.1 |
| GMI, % | 7.3 ± 0.9 |
| Time in range, % | 59.0 ± 15.9 |
| Time below range, % | 5.7 ± 5.2 |
| Time below 54mg/dL, % | 1.5 ± 2.4 |
| Time above range, % | 36.5 ± 20.1 |
| Time above 250mg/dL, % | 13.5 ± 15.3 |
| CV, % | 38.3 ± 6.0 |

| Supplemental table 2b |  |
| --- | --- |
| Without statin, n (%) | 67 (74.4%) |
| With statin, n (%) | 23 (25.6%) |
| Atorvastatin, n (%) | 19 (21.1%) |
| Rosuvastatin, n (%) | 3 (3.3%) |
| Simvastatin, n (%) | 1 (1.1%) |
| Statin Potency |  |
| Low, n (%) | 1 (4.3%) |
| Moderate, n (%) | 13 (56.5%) |
| High, n (%) | 9 (39.1%) |
| With Ezetimibe | 5 (5.6%) |
| Total cholesterol, mg/dL | 165.8 ± 36.3 |
| HDL cholesterol, mg/dL | 58.0 ± 16.2 |
| LDL cholesterol, mg/dL | 91.6 ± 30.1 |
| Triglycerides, mg/dL | 79.6 ± 38.1 |
| Non-HDL cholesterol, mg/dL | 107.6 ± 30.5 |

**Supplemental table 2 caption:**

Baseline characteristics of the longitudinal analysis (n=90).

**Supplemental table 2a:** Baseline characteristics of the participants included in the longitudinal analysis such as demographic and social features and T1D monitoring values.

**Supplemental table 2b:** Baseline characteristics the participants included in the longitudinal analysis such as statin treatment and lipid profile analysis.

ASCVD: Atherosclerotic Cardiovascular Disease; HbA1C: Hemoglobin A1C; GMI: Glucose management indicator; CV: Coefficient of variability; LDL: Low-density lipoprotein; HDL: High-density lipoprotein.
