# Supplementary material for: Association of glycemic variability and time in range with lipid profile in type 1 diabetes
Source: Endocrine. 2023 Dec 5;83(1):69–76. doi: 10.1007/s12020-023-03464-x (PMC10805887; doi:10.1007/s12020-023-03464-x)
Supplement: Supplementary file 4 — Supplemental table 3 [file 12020_2023_3464_MOESM4_ESM.docx]

**Supplemental table 3**

| Supplemental table 3a | Without insulin pump,  n=157 | With insulin pump,  n=85 |  |
| --- | --- | --- | --- |
| Male sex, n (%) | 53.2 | 48.8 | p=0.52 |
| Age, years | 38.1 ± 12.8 | 33.9 ± 11.7 | p=0.13 |
| Educational level, n (%) |  |  | **p=0.001** |
| Less than 9^th^ grade | 15.2 | 3.9 |  |
| 9^th^ to 12^th^ grade | 37.0 | 23.4 |  |
| Higher Education | 47.8 | 72.7 |  |
| Duration of diabetes, years | 15.4 ± 10.4 | 19.2 ± 9.6 | **p=0.007** |
| Body Mass Index, kg/m^2^ | 24.9 ± 3.9 | 24.5 ± 3.9 | p=0.55 |
| Physical activity, n (%) | 43.1 | 52.2 | p=0.22 |
| With smoking habits, n (%) | 31.1 | 8.5 | p=0.001 |
| With drinking habits, n (%) | 15.1 | 6.9 | p=0.12 |
| Hypertension, n (%) | 14.6 | 10.6 | p=0.37 |
| ASCVD, n (%) | 5.7 | 3.5 | p=0.45 |
| Nephropathy, n (%) | 7.0 | 11.8 | p=0.21 |
| Retinopathy, n (%) | 24.2 | 18.3 | p=0.30 |
| Neuropathy, n (%) | 3.8 | 4.8 | p=0.73 |
| Heart failure, n (%) | 2.6 | 1.2 | p=0.48 |
| HbA1C, % | 7.8 ± 1.5 | 7.4 ± 0.9 | **p=0.020** |
| GMI, % | 7.5 ± 1.0 | 7.3 ± 0.8 | p=0.15 |
| Time in range, % | 56.0 ± 19.0 | 58.1 ± 16.7 | p=0.40 |
| Time below range, % | 6.2 ± 7.9 | 7.3 ± 0.8 | p=0.27 |
| Time below 54mg/dL, % | 2.1 ± 4.0 | 2.6 ± 4.6 | p=0.26 |
| Time above range, % | 44.4 ± 28.2 | 40.3 ± 23.6 | p=0.26 |
| Time above 250mg/dL, % | 16.5 ± 16.4 | 13.4 ± 11.9 | p=0.13 |
| CV, % | 38.2 ± 8.1 | 39.0 ± 6.9 | p=0.45 |
| Waist circunference, cm | 85.7 ± 14.3 | 84.5 ± 13.0 | p=0.82 |
| TDD, IU | 51.8 ± 24.3 | 52.5 ± 16.9 | p=0.88 |
| TDD/kg, IU/kg | 0.7 ± 0.3 | 0.8 ± 0.2 | p=0.57 |

| Supplemental table 3b | Without insulin pump,  n=157 | With insulin pump,  n=85 |  |
| --- | --- | --- | --- |
| Total cholesterol, mg/dL | 168.0 ± 36.4 | 163.7 ± 38.7 | p=0.41 |
| HDL cholesterol, mg/dL | 56.2 ± 12.7 | 59.3 ± 38.7 | p=0.13 |
| LDL cholesterol, mg/dL | 97.4 ± 30.8 | 91.0 ± 26.0 | p=0.13 |
| Triglycerides, mg/dL | 83.7 ± 40.5 | 73.6 ± 43.9 | p=0.08 |
| Non-HDL cholesterol, mg/dL | 112.3 ± 31.5 | 104.5 ± 31.2 | p=0.08 |

**Supplemental table 3 caption:**

Comparison of the baseline characteristics of participants with and without an insulin pump (n= 157 and n=85, respectively).

**Supplemental table 3a:**

Comparison of the demographic and social features and T1D monitoring values of participants with and without insulin pump.

**Supplemental table 3b:**

Comparison of lipid profile analysis of participants with and without an insulin pump.

ASCVD: Atherosclerotic Cardiovascular Disease; HbA1C: Hemoglobin A1C; GMI: Glucose management indicator; CV: Coefficient of variability; TDD: Total Daily Dose; LDL: Low-density lipoprotein; HDL: High-density lipoprotein; T1D: Type 1 Diabetes.
