# Supplementary material for: Association of glycemic variability and time in range with lipid profile in type 1 diabetes
Source: Endocrine. 2023 Dec 5;83(1):69–76. doi: 10.1007/s12020-023-03464-x (PMC10805887; doi:10.1007/s12020-023-03464-x)
Supplement: Supplementary file 5 — Supplemental table 4 [file 12020_2023_3464_MOESM5_ESM.docx]

**Supplemental table 4**

| Supplemental table 4a | BMI < 30 kg/m^2^, n=177 | BMI ≥ 30 kg/m^2^, n=20 |  |
| --- | --- | --- | --- |
| Male sex, n (%) | 50.6 | 68.4 | p=0.14 |
| Age, years | 39.9 ± 12.4 | 39.7 ± 12.4 | p=0.14 |
| Educational level, n (%) |  |  | **p=0.001** |
| Less than 9^th^ grade | 11.4 | 0.0 |  |
| 9^th^ to 12^th^ grade | 30.7 | 73.7 |  |
| Higher Education | 57.8 | 26.3 |  |
| Duration of diabetes, years | 16.2 ± 9.9 | 17.5 ± 11.3 | p=0.57 |
| With insulin pump, n (%) | 35.7 | 28.0 | p=0.44 |
| Physical activity, n (%) | 46.3 | 47.4 | p=0.93 |
| With smoking habits, n (%) | 21.3 | 31.6 | p=0.31 |
| With drinking habits, n (%) | 10.8 | 22.2 | p=0.16 |
| Hypertension, n (%) | 10.7 | 30.0 | **p=0.014** |
| ASCVD, n (%) | 4.0 | 15.0 | **p=0.014** |
| Nephropathy, n (%) | 11.3 | 5.0 | p=0.39 |
| Retinopathy, n (%) | 23.4 | 15.0 | p=0.72 |
| Neuropathy, n (%) | 3.4 | 5.0 | p=0.72 |
| Heart failure, n (%) | 0.6 | 5.0 | p=0.06 |
| HbA1C, % | 7.6 ± 1.4 | 7.8 ± 0.8 | p=0.56 |
| GMI, % | 7.4 ± 0.9 | 7.6 ± 0.6 | p=0.34 |
| Time in range, % | 57.5 ± 18.8 | 52.6 ± 0.6 | p=0.25 |
| Time below range, % | 6.6 ± 8.1 | 5.5 ± 8.4 | p=0.55 |
| Time below 54mg/dL, % | 2.2 ± 4.3 | 2.0 ± 4.2 | p=0.87 |
| Time above range, % | 41.4 ± 26.2 | 51.4 ± 20.4 | p=0.10 |
| Time above 250mg/dL, % | 14.8 ± 15.1 | 18.9 ± 12.5 | p=0.25 |
| CV, % | 38.3 ± 8.0 | 39.5 ± 6.9 | p=0.52 |
| Waist circunference, cm | 82.7 ± 11,9 | 107.3 ± 6.4 | **p=0.002** |
| TDD, IU | 50.8 ± 19.9 | 165.2 ± 32.3 | p=0.07 |
| TDD/kg, IU/kg | 0.7 ± 0.3 | 0.7 ± 0.4 | p=0.96 |

| Supplemental table 4b | BMI < 30 kg/m^2^, n=177 | BMI ≥ 30 kg/m^2^, n=20 |  |
| --- | --- | --- | --- |
| Total cholesterol, mg/dL | 165.0 ± 38.3 | 16.2 ± 32.3 | p=0.95 |
| HDL cholesterol, mg/dL | 58.0 ± 14.8 | 49.1 ± 9.8 | **p=0.014** |
| LDL cholesterol, mg/dL | 94.3 ± 28.6 | 93.7 ± 29.0 | p=0.94 |
| Triglycerides, mg/dL | 75.9 ± 37.0 | 115.8 ± 56.4 | **p=0.001** |
| Non-HDL cholesterol, mg/dL | 108.0 ± 32.0 | 116.1 ± 28.2 | p=0.31 |

**Supplemental table 4 caption:**

Comparison of the baseline characteristics between participants with normal BMI and with obesity (BMI ≥ 30 kg/m2) (n= 177 and n=20, respectively).

**Supplemental table 4a:**

Comparison of the demographic and social features and T1D monitoring values between participants with normal BMI and with obesity.

**Supplemental table 4b:**

Comparison of lipid profile analysis between participants with normal BMI and participants with obesity.

BMI: Body Mass Index; ASCVD: Atherosclerotic Cardiovascular Disease; HbA1C: Hemoglobin A1C; GMI: Glucose management indicator; CV: Coefficient of variability; TDD: Total Daily Dose; LDL: Low-density lipoprotein; HDL: High-density lipoprotein; T1D: type 1 diabetes.
